# The Endocannabinoid System in the Retina: From Physiology to Practical and Therapeutic Applications

**DOI:** 10.1155/2016/2916732

**Published:** 2016-01-06

**Authors:** Thomas Schwitzer, Raymund Schwan, Karine Angioi-Duprez, Anne Giersch, Vincent Laprevote

**Affiliations:** ^1^Pôle Hospitalo-Universitaire de Psychiatrie, Centre Psychothérapique de Nancy, Laxou, France; ^2^EA7298, INGRES, Université de Lorraine, Vandœuvre-lès-Nancy, France; ^3^INSERM U1114, Fédération de Médecine Translationnelle de Strasbourg, Département de Psychiatrie, Centre Hospitalier Régional Universitaire de Strasbourg, Strasbourg, France; ^4^Maison des Addictions, CHU Nancy, Nancy, France; ^5^Service d'Ophtalmologie, CHU Nancy, Nancy, France

## Abstract

*Cannabis* is one of the most prevalent drugs used in industrialized countries. The main effects of* Cannabis* are mediated by two major exogenous cannabinoids: ∆9-tetrahydroxycannabinol and cannabidiol. They act on specific endocannabinoid receptors, especially types 1 and 2. Mammals are endowed with a functional cannabinoid system including cannabinoid receptors, ligands, and enzymes. This endocannabinoid signaling pathway is involved in both physiological and pathophysiological conditions with a main role in the biology of the central nervous system. As the retina is a part of the central nervous system due to its embryonic origin, we aim at providing the relevance of studying the endocannabinoid system in the retina. Here, we review the distribution of the cannabinoid receptors, ligands, and enzymes in the retina and focus on the role of the cannabinoid system in retinal neurobiology. This review describes the presence of the cannabinoid system in critical stages of retinal processing and its broad involvement in retinal neurotransmission, neuroplasticity, and neuroprotection. Accordingly, we support the use of synthetic cannabinoids as new neuroprotective drugs to prevent and treat retinal diseases. Finally, we argue for the relevance of functional retinal measures in cannabis users to evaluate the impact of cannabis use on human retinal processing.

## 1. Introduction

Cannabis is one of the most prevalent drugs used worldwide. The main constituents of cannabis are Δ9-tetrahydrocannabinol (THC) and cannabidiol (CBD) [1–3]. They act on specific cannabinoid receptors, mainly cannabinoid receptors type 1 and 2, CB1, and CB2 [4–8]. Although it is still debated whether to consider the G protein-coupled receptor 55 (GPR55) as a cannabinoid receptor, several evidences suggest that it might also be a cannabinoid receptor [9]. Beside the effects caused by exogenous cannabinoids, CB1 and CB2 are stimulated by two major endogenous ligands: N-arachidonoylethanolamide (anandamide, AEA) and 2-arachidonoylglycerol (2-AG) [10, 11]. Membrane phospholipids are metabolized by calcium-dependent phospholipases to release AEA and 2-AG (for a review, see [12]). Briefly, the precursor of AEA is N-arachidonoyl phosphatidylethanolamine (NAPE) which is hydrolyzed by a phospholipase D to release AEA and phosphatidic acid. 2-AG synthesis is based on hydrolysis of diacylglycerols (DAG) by two DAG-lipase isozymes, DAGL*α* and DAGL*β*. The cellular level of these ligands is regulated by several enzymes, especially fatty acid amide hydrolase (FAAH), monoacylglycerol lipase (MGL), and cyclooxygenase-2 (COX-2) [13]. Together, cannabinoid receptors, ligands, and enzymes constitute a functional cannabinoid system involved throughout the body in several physiological mechanisms as well as in pathophysiological conditions. Importantly, this system is implicated in the regulation of the central nervous system (CNS) neurobiology. Interestingly, the cannabinoid system plays a major role in the regulation of CNS neurotransmission [14, 15]. As CB1 receptors have predominantly a presynaptic localization in the brain neurons, they play a postsynaptic regulatory role by modulating the release of several neurotransmitters such as gamma-aminobutyric acid (GABA), glutamate, and dopamine [16–18]. For example, the stimulation of a glutamatergic neuron results in a synaptic release of glutamate, which induces a postsynaptic influx of calcium through NMDA receptors [14]. Consequently, the process of synaptic strengthening is activated by this increase of the calcium concentration [14]. Afterward, there is a postsynaptic synthesis of endocannabinoids by the stimulation of postsynaptic metabotropic glutamate receptors, mGlu. Through CB1 presynaptic receptors, endocannabinoids regulate the presynaptic glutamate release and avoid any excessive postsynaptic release of calcium [13, 14]. Exogenous cannabinoids, by binding to the CB1 receptors, disrupt the regulation of glutamate release mediated by endocannabinoids. An excess of postsynaptic influx of calcium occurs, thus accelerating the pruning synaptic process and the apoptosis of the cell [13, 14]. In the CNS, cannabinoid ligands can also act on postsynaptic CB1 receptors. For example, through postsynaptic CB1 receptors they regulate the slow self-inhibition of neocortical interneurons and pyramidal neurons, an endocannabinoid dependent persistent change of somatodendritic excitability [19].

**Figure 1:**
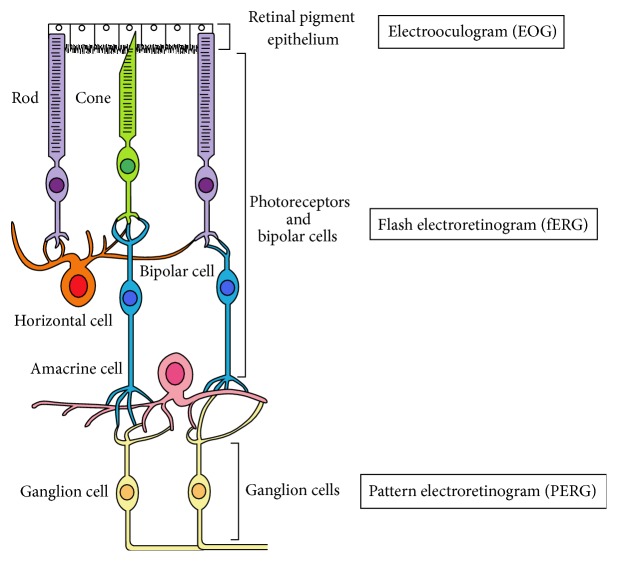
Schematic representation of the retina and standardized electrophysiological methods allowing the assessment of the retinal function.

The retina is anatomically and developmentally an extension of the CNS, and the retina and the brain are connected by the optic nerve, the axons of the ganglion cells, through the lateral geniculate nucleus [20]. The retina and the brain present similar properties. They express several neurotransmitters such as dopamine [21, 22], serotonin [23], glutamate, and GABA [24, 25]. Retinal processing, as measured by electrophysiological measurements (flash electroretinogram (fERG), pattern electroretinogram (PERG), and electrooculogram (EOG)) is sensitive to pharmacological drugs acting on the CNS neurotransmission [26–29]. Finally, CNS disorders, such as neurological, psychiatric, and addictive diseases, display manifestations in the retina [15, 30–34]. Briefly, the retina represents the neural portion of the eye and is composed of several neuronal cell layers (Figure 1). The light penetrates the eye through the anterior eye structure [20]. Then, it reaches the outer segment of the retina where the photoreceptor cells are located, namely, rods and cones [20]. At this level, the light is absorbed by the photopigments of the photoreceptors, thus initiating the conversion of light into an electric signal, called the phototransduction process [20]. This signal is transferred to the bipolar cells and then to the ganglion cells, the axons of which form the optic nerve, which transfers the visual information to the visual cortex via the lateral geniculate nucleus. This signal is also under the influence of interneuron cells, namely, amacrine and horizontal cells [20]. Moreover, the retina contains Müller cells acting as a glia [20]. The retinal pigment epithelium is also part of the retina and plays a role in several trophic functions such as light absorption, photoreceptor disk renewal, and immune modulation, to name a few [20, 35].

In the CNS, the manipulation of endocannabinoids has been reported to attenuate brain damages induced by a variety of insults. Moreover, the cannabinoid system signaling pathways is considered as a critical process involved in neuron survival. For instance, an upregulation of the endocannabinoid system is known as an adaptive response that attenuates CNS damages in the context of trauma and neurodegenerative diseases [36, 37] and is considered as a neuroprotective strategy against neuropathological states [38]. In line with these observations, the inhibition of endocannabinoid degradation induces a decrease in pathological tissues in experimental conditions of CNS injuries [39]. As the retina is considered as a part of the CNS, it is now crucial to assess whether the cannabinoid system is distributed in the retina and is involved in retinal pathological or retinal protective conditions.

The cannabinoid system has been detected in ocular tissues and other critical stages of visual information processing. In ocular tissues, CB1 receptors have been localized in the ciliary body of rat, bovine, and human and in the trabecular meshwork in bovine and human [40–42]. CB1 receptors were also found in the nonpigmented ciliary epithelium in human and bovine and in the conjunctival epithelium in mouse and human [42–44]. Hydrolysis of anandamide was detected in the porcine iris, choroid, lacrimal gland, and optic nerve [45]. AEA and 2-AG were expressed in human cornea, ciliary body, iris, and choroid [46, 47]. Endocannabinoids are also found in later and more integrated stages of visual processing, especially in the lateral geniculate nucleus (LGN) and in the visual brain (for a review see [15]). Briefly, CB1 receptors are found in the LGN, superior colliculus, and suprachiasmatic nucleus in rat and mouse and in the LGN and the superior colliculus in human. CB1 and CB2 are expressed in the primary (V1) and secondary (V2) visual cortex of rat and mouse. In nonhuman primate and human, CB1 receptors are detected in V1 and V2 [15].

This review focuses on the neurobiological role of the cannabinoid system in the retina. We first review the distribution of the cannabinoid receptors, ligands, and enzymes in the retina. Then, we review studies that have examined the role of the cannabinoid system in retinal neurotransmission, neuroplasticity, and neuroprotection. Based on these results, we argue for the development of synthetic cannabinoids as therapeutic agents for the treatment and the prevention of retinal diseases. Finally, we support potential effects of cannabis use on human retinal processing and we present several functional measurements allowing the rigorous assessment of the retinal function in cannabis users.

## 2. Materials and Methods

In order to thoroughly explore the role of the cannabinoid system in the retina, a search for relevant articles was conducted in the PubMed, ScienceDirect, and Google Scholar databases using the following keywords: (“cannabinoid system” or “endocannabinoids” or “cannabinoids” or “cannabinoid ligands” or “cannabinoid receptors” or “cannabinoid enzymes”) and (“neurotransmission” or “neuroplasticity” or “neuroprotection”) and (“retina” or “retinal”). All results up to June 1, 2015, were examined for the selection process. Relevant publications were chosen through an individual independent selection of titles by the following authors: Thomas Schwitzer, Raymund Schwan, Anne Giersch, and Vincent Laprevote. The articles selected had to be written in English and be related to the topic of the review. Additionally, a manual search was performed on the bibliography of each selected article.

## 3. Endocannabinoids in the Retina: Receptors, Ligands, and Enzymes

### 3.1. Retinal Endocannabinoid Receptors

Numerous studies have shown the presence of the cannabinoid receptors CB1 and CB2 in the retina of several animal species such as tiger salamander, goldfish, rat, mouse, chick, and monkey [41, 48–58]. CB1 receptors are detected in inner and outer plexiform layers and two synaptic layers of the retina of all of these species [56]. CB1 receptors are also expressed in the main cells of the neural retina, especially in the cone pedicles and rod spherules of photoreceptors of the same species [56]. Furthermore, they are found in horizontal cells, amacrine cells, ganglion cells, and ganglion cell axons of these species except goldfish [53, 56]. In monkey, rat, mouse, and chick, CB1 receptors are detected in inner and outer segments of the photoreceptors [56]. In monkey, CB1 receptors are expressed throughout the retina from the fovea to the retinal periphery and in particular in cones of the central retina [49]. In goldfish, CB1 receptors are mainly localized intracellularly and on the plasma membrane of photoreceptors, bipolar cells, and amacrine cells [57]. CB1 receptors are also found in Müller cells and in synaptic terminal of On and Off bipolar cells in goldfish [57].

Although the presynaptic location of CB1 receptors was readily described, especially in gabaergic and glutamatergic neurons, there are several evidences also indicating a postsynaptic localization. For example, Chaves et al. showed that retinal ablation induced an increase in level of CB1 proteins in the optic tectum and other retinorecipient visual areas in the adult chick brain with no change in CB1 mRNA levels [59]. This increase in CB1 receptors expression after retinal ablation suggests a postsynaptic location of these receptors in the retinotectal axons.

CB2 receptors are detected in the retina of several species. In the rat retina, CB2 receptors are expressed in photoreceptors, horizontal cells, amacrine cells, inner nuclear layer, inner plexiform layer, retinal pigment epithelium, and retinal ganglion cell layer, especially in the somas of retinal ganglion cells [54, 55]. In mouse, CB2 receptors are localized in cone and rod photoreceptors, horizontal cells, amacrine cells, bipolar cells, and ganglion cells [51]. CB2 receptors are also detected in Müller cells in monkey [48] and in goldfish [52].

In addition, CB1 and CB2 receptors are expressed in the human retina. CB1 receptors are detected in outer segments of photoreceptor cells, inner plexiform layer, outer plexiform layer, two synaptic layers of the retina, inner nuclear layer, ganglion cell layer, and retinal pigment epithelium cells [40, 43, 60]. CB2 receptors are expressed in human retinal pigment epithelium cells [60].

### 3.2. Retinal Endocannabinoid Ligands

The two main endogenous cannabinoid ligands acting on cannabinoid receptors, namely, anandamide and 2-AG, are found in the retina of tiger salamander, goldfish, rat, mouse, chick, bovine, porcine, and monkey [45, 56, 61, 62]. In human, 2-AG is expressed at a high level in the retina [46, 47], whereas anandamide is detected at a lower level in the retina [42, 46, 47].

### 3.3. Retinal Endocannabinoid Enzymes

The cellular level of retinal endocannabinoids is regulated by several main enzymes: FAAH, MGL, and COX-2. These enzymes are detected in the retina and enable the degradation of cannabinoid ligands [13]. FAAH is an integral membrane protein and is expressed throughout the monkey retina, from the fovea to the retinal periphery [49]. Most specifically, FAAH is detected in photoreceptors, outer plexiform layer, inner nuclear, inner plexiform layer, and retinal ganglion cell layer in monkey [49]. Concerning photoreceptors in monkey retina, FAAH is preferentially expressed in cones of the central retina and is mainly located in cone pedicles and rod spherules [49]. FAAH is also detected in cone and rod bipolar cells and in ganglion cell somas and axons in the monkey retina [49]. In the rat, goldfish, and bovine retina, a FAAH like activity is detected [61–63]. In the rodent retina—mouse and rat—a FAAH activity is detected in rods, bipolar cells, horizontal cells, amacrine cells, Müller cells, and ganglion cells [63]. In goldfish, FAAH is most prominent over Müller cells and cone inner segments and is observed at a lower level in amacrine cells, cell bodies in the ganglion cell layer, and the inner plexiform layer [62]. FAAH is also expressed in the human retina, in particular in retinal pigment epithelium cells [60].

Preliminary findings are consistent with the detection of MGL in inner plexiform layer, rod bipolar cells, amacrine cells, and ganglion cells in the mouse retina [13, 64]. Similarly, the presence of COX-2 has been shown in horizontal cells, amacrine cells, ganglion cells, and Müller cells in the rat retina [65] whereas COX-2 has been detected in rod and bipolar cells in the mouse retina [13]. Recent findings report a detection of COX-2 in the human retina [66].

Enzymes allowing the synthesis of cannabinoid ligands are also expressed in the retina [64, 67]. For instance, NAPE responsible for the synthesis of AEA was identified in the bovine retina by means of gas chromatography-electron impact mass spectrometry [61]. The enzyme responsible for the synthesis of 2-AG named DAGL*α* has been detected in the two synaptic layers, the outer plexiform layer, and the inner plexiform layer of the mouse retina [64]. Importantly, DAGL*α* is localized in postsynaptic terminals of type 1 OFF cone bipolar cells whereas the expression of DAGL*β* appears to be restricted to retinal blood vessels in the mouse retina [64]. DAGL*α* is detected early in postnatal development in the rat retina in photoreceptors—cones and rods—cone bipolar cells, horizontal, amacrine, and ganglion cells [67].

The endocannabinoid system is detected in critical stages of retinal information processing such as photoreceptors, bipolar cells, and ganglion cells. These findings support a role of endocannabinoids in the modulation of retinal neurobiology as well as in the regulation of vertical transmission of the retinal information.

## 4. Neurobiology of Cannabinoids in the Retina: Neurotransmission, Neuroplasticity, and Neuroprotection

### 4.1. Neurotransmission

Neurotransmission is characterized by the transmission of a nerve impulse across a synapse. The modulation of ionic channels or enzymatic activity, to name a few, can affect neurotransmission. Previous studies have outlined the involvement of the cannabinoid system in these mechanisms, thus allowing the regulation of the retinal neurotransmission [13, 15].

Several inward and outward ionic channels are known to play a major role in retinal physiology [20]. For example, sodium, calcium, chloride, and potassium channels are involved in the phototransduction process and especially in the depolarization and the hyperpolarization of photoreceptor and bipolar cells [20]. Different studies have shown that cannabinoid agonists induced a dose-dependent reversible modulation of calcium, potassium, and chloride currents in bipolar, rod, cone, and ganglion cells [53, 56, 57, 68–74]. These findings suggest a regulatory role of the cannabinoid system in the retinal neurotransmission at the level of photoreceptor, bipolar, and ganglion cells, which constitute three critical stages of the neural retina. As a consequence, stimulation of the cannabinoid system may modulate the vertical transmission of the retinal information and consequently may alter visual perception.

A direct action of cannabinoids on retinal enzymatic activity or retinal transmitter release has also been described [72, 75–79]. In the bovine retina, THC has led to a dose-dependent regulation of monoamine oxidase activity thus altering the retinal neurotransmission [75]. Similarly, in the isolated bovine retina, cannabinoid CB1 receptor agonists, but not CB2 agonists, inhibited aspartate release, which was blocked by cannabinoid antagonists [72]. In perfused guinea-pig retinal discs, dopamine and noradrenaline transmission release was inhibited by activation of cannabinoid receptors CB1, which was blocked by cannabinoid antagonists [77, 79]. Of interest, the release of several retinal neurotransmitters such as dopamine, noradrenaline, GABA, and glutamate is therefore modulated by cannabinoids [72, 73, 76–79].

Finally, studies on the goldfish retina argue for a role of the cannabinoid system in the regulation of the phototransduction cascade, the dark and light retinal sensitivity, the dark and light retinal adaptation, and the retinal contrast sensitivity [57, 70, 80].

### 4.2. Neuroplasticity

The role of the cannabinoid system in the regulation of short-term and long-term plasticity in the CNS has been readily described [81–84]. However, few studies have investigated the effects of cannabinoids on retinal synaptic plasticity. The messenger ribonucleic acid (mRNA) for cannabinoid receptors has been detected during development, especially in the embryonic and adult rat retina [50, 55]. Recent findings suggest that exogenous cannabinoids may alter both synaptic transmission and synaptic plasticity in the retina, in particular in retinal ganglion cells [76]. Using whole-cell voltage-clamp recordings in retinal ganglion cells in adult and young mice (P14–P21), the administration of an exogenous cannabinoid agonist significantly reduced the frequency of spontaneous postsynaptic currents (SPSCs) in these cells [76]. This change did not modify the kinetics of the spontaneous postsynaptic currents. Consequently, it was suggested that the cannabinoid agonist had a presynaptic action and that it could decrease the release of both GABA and glutamate. As the largest effect was found in young mice and was different from adult mice, these results argue for a developmental role of the cannabinoid system in the maturation of synaptic retinal circuits [76].

### 4.3. Neuroprotection

Neuroprotection is a protecting mechanism that consists in preventing the death of damaged neurons and their degeneration, due to a hostile environment created by an initial cellular stress. Using several models of retinal diseases, mounting evidence suggests that the retinal cannabinoid system might play a neuroprotective role in the retina. Oxidative stress is known to be a key mechanism in the pathological process of age-related macular degeneration (AMD) and diabetic retinopathy (DR) [85, 86]. In AMD, a trend towards an increase in retinal anandamide level was observed [47]. In a cellular model of AMD, an inhibition of CB1 receptors protected retinal pigment epithelium cells from oxidative damage [87]. The DR is characterized by an oxidative stress, a breakdown of the blood-retinal barrier, and a proinflammatory effect to name a few, which are associated with retinal neuronal death [85]. A recent study showed that the retinal concentration of anandamide was increased in DR whereas no change in the retinal level of 2-AG was observed [47]. In a rat model of DR, treatment with cannabidiol significantly reduced both oxidative stress and neurotoxicity and prevented retinal cell death [88]. Consequently, exocannabinoids may be a relevant therapeutic strategy decreasing oxidative stress signaling and preventing neurodegeneration of retinal cells in AMD and DR. The neuroprotective role of cannabinoids was also shown in an animal model for autosomal dominant retinitis pigmentosa (RP) on the photoreceptor degeneration, synaptic connectivity, and functional activity of the retina [89]. In this rat model, the administration of a synthetic cannabinoid agonist from P24 to P90 induced improvements of visual function compared to vehicle-administered animals [89]. In fact, the enhancement of vision loss was demonstrated by increased electroretinogram signals, especially scotopic a- and b-wave amplitudes [89]. These changes were correlated with a delay in the degeneration of photoreceptors and with the preservation of presynaptic and postsynaptic elements. These crucial findings support the retinal protective role of exocannabinoids on both the structural and functional properties of retinal cells. Retinal ganglion cell death may be a consequence of a neurodegenerative retinal disease such as glaucoma. An excessive extracellular glutamate release has been identified as one of the pathophysiological mechanisms inducing excitotoxicity in glaucoma via the excessive formation of peroxynitrite [90]. The neuroprotective effect of THC and cannabidiol was observed through the limitation of peroxynitrite production in a rat model of excitotoxicity consisting in intravitreal injection of N-methyl-D-aspartate (NMDA) [91]. Similarly, weekly injections of THC decreased intraocular pressure and reduced the loss of retinal ganglion cells in the peripheral and central retina [92]. In another model of intraocular pressure, an ischemic-reperfused retina model, systemic administrations of a FAAH inhibitor decreased enzymatic activity and consequently reduced the retinal damage caused by the ischemic-reperfusion mechanism [93]. Additionally, intravitreal injections of an AEA agonist reduced retinal ganglion cell loss which was abolished by the systemic administration of a CB1 antagonist [93]. In another example, the administration of an inhibitor of FAAH increased retinal ganglion cell survival following optic nerve axotomy in young and aged rats [94]. This effect was affected by a cannabinoid antagonist and was associated with both an increase in anandamide and a decrease in anandamide metabolites, with no effect on 2-AG level. All these results are in accordance with the relevance of local or systemic administrations of exocannabinoids in the prevention of retinal ganglion cell loss due to retinal diseases. Finally, anandamide and 2-AG were also involved in the modulation of the innate immune response in human retinal Müller glia to combat inflammation in the retina during human immunodeficiency virus (HIV) infection [95]. The HIV infection induces a retinal neurodegeneration by increasing the inflammatory response, which consequently causes retinal impairments. In this case, both anandamide and 2-AG induced a decrease in the inflammation and limited the retinal loss [95]. These data argue for the use of cannabinoids in retinal diseases as new therapeutic agents to prevent neurodegeneration and cell death.

## 5. Cannabis Use and Human Retina

According to the large distribution of endocannabinoids in the retina and considering the role of cannabinoids in modulating the retinal neurophysiology, it is now crucial to consider potential effects of cannabis, after both acute and regular use, on the structural and functional characteristics of the human retina [15, 31]. The retina is organized in cell layers of which functional properties can be assessed by electrophysiological techniques (Figure 1). The electroretinogram (ERG) measurements, the fERG, and the PERG allow the assessment of the functional properties of specific cell types in the neural retina [96, 97]. ERG measurements are objective and noninvasive techniques as well as rapid and costless methods. The retina also contains the retinal pigment epithelium (RPE). The functioning of the RPE can be assessed by the EOG. Each one of these techniques is described below.

The fERG measures the electric biopotential evoked mainly by photoreceptor cells, namely, rods and cones, and the ON-bipolar and Müller cells complex, in response to a light stimulation. fERG recordings performed under photopic and scotopic conditions are called light- and dark-adapted fERG, respectively, according to the flash luminance intensity used, which is measured in candelas·s·m^−2^ (cds·s·m^−2^) [97]. Two main components are usually observed on a typical fERG trace: an electronegative component called a-wave, followed by an electropositive component named b-wave. The a-wave is generated by the hyperpolarization of the photoreceptors and the b-wave reflects the depolarization of the ON-bipolar and Müller cells complex. Although the a-wave and the b-wave are commonly the most analyzed, other components are detected in the full-field ERG such as the photopic negative response (PhNR). This response is a negative component that follows the b-wave and reflects the activity of innerretinal cells, especially the ganglion cells [98]. Specific conditions are needed to easily detect the PhNR. Indeed, a brief red flash against a blue background is an optimal configuration for eliciting this response [99].

The PERG records the central macular function of the retina, as well as the retinal ganglion cells response using reversal black and white checkerboards viewed with a central fixation [100]. To investigate the transient PERG, checkerboards are presented at 2–6 reversals per second (1–3 Hz). Two main waves are usually described on the transient PERG trace: a positive wave named P50, followed by a negative wave called N95 [101]. Like the fERG, both the amplitude and implicit time of these waves can be measured [96]. P50 is, in part, attributed to the retinal ganglion cells and macular photoreceptors and is used to evaluate the macular function. N95 is generated by the retinal ganglion cells and reflects their functioning [102].

The EOG measures a variation of electrical potentials between skin electrodes located on the external and internal canthi [103]. This variation corresponds to an electrical potential between the front and the back of the eye and is called standing potential [104]. This potential mainly originates from the retinal pigment epithelium and varies with the retinal illumination. It is obtained by asking the subjects to make lateral eye movements in both photopic and scotopic conditions. It is possible to derive two parameters from the EOG trace, namely, the dark trough, which represents the trough of the curve in dark condition whose origin remains unclear, and the light peak, which represents the maximal peak in light condition and corresponds to the maximal depolarization of the basal membrane of the retinal pigment epithelium.

The neural retina is composed by three critical stages: the photoreceptor cells, called rods and cones, the bipolar cells, and the ganglion cells. The retina also contains interneuron cells: amacrine and horizontal cells and the retinal pigment epithelium. The functional properties of photoreceptors, bipolar cells, ganglion cells, and retinal pigment epithelium cells are evaluated by objective electrophysiological retinal techniques.

Considering the large distribution of the cannabinoid system in the different retinal cell layers and its broad involvement in the regulation of retinal neurophysiology, these electrophysiological measures may be of benefit to the evaluation of the impact of cannabis on the human retinal function.

To date, only one study, to our knowledge, has evaluated the impact of cannabis on the retinal function [105]. In this study, no fERG abnormality was found neither in a man suffering for hallucinogen persisting perception disorder after marijuana consumption nor in four heavy cannabis smokers with no visual disturbance [105]. However, several EOG anomalies were observed in the patient with hallucinogen perception showing effects of cannabis on the retinal pigment epithelium functioning. The small number of subjects and the absence of control group probably explain the lack of ERG alterations.

Although cannabis is one of the most prevalent drugs used worldwide, there is to date few studies that have evaluated the impact of cannabis on the human visual function. These studies were already reported in several reviews [13, 15]. As discussed in these reviews, there is no certainty that the visual abnormalities found in cannabis users originated from the retina because no studies evaluating the retinal structural and functional properties have yet been performed in cannabis users. However, some hypothesis could explain the impact of cannabis on human retinal processing. Cannabis is a neurotoxic and neuromodulator substance that acts on several inhibitory and excitatory neurotransmitters signaling pathways in the CNS. The main effects of cannabis concern the glutamatergic, gabaergic, and dopaminergic brain synaptic transmissions [14]. All of these neurotransmitters are detected in the retina and play several key roles in retinal physiology. For instance, dopamine is the main catecholamine expressed in the human retina and is known to be involved in light adaptation [22]. As another example, glutamate and GABA are two amino-acid neurotransmitters expressed in the retina and are involved in numerous regulatory mechanisms in the retina, especially those concerning the synaptic transmission [24, 25]. In addition, glutamate plays a major role in the vertical transmission of the retinal signal [24, 25]. Accordingly, cannabis could disrupt the regulatory role of the cannabinoid system in the retina and consequently alter the transmission of the retinal information. As a consequence, studies evaluating the impact of cannabis on human retinal function are genuinely needed [15, 31].

## 6. Discussion

This review outlines the presence of endocannabinoids in critical stages of the neural retina such as photoreceptor cells, bipolar cells, and ganglion cells. Endocannabinoids are also detected in interneuron cells, namely, amacrine and horizontal cells, as well as in Müller and retinal pigment epithelium cells. The presence of endocannabinoids in the retina supports a regulatory role of the cannabinoid system in the vertical transmission of visual information from photoreceptors to ganglion cells, the ultimate stage before the transmission of the visual information to the brain, mostly the visual cortex. This also supports a potential dysregulation induced by exogenous cannabinoids, such as THC and cannabidiol, the main constituents of cannabis. This review also described the localization of the cannabinoid system in the retinal pigment epithelium suggesting a role of this system in the renewal of the photoreceptor disks as well as in trophic functions. Another important finding of this review is the involvement of cannabinoids in retinal neurotransmission, neuroplasticity, and neuroprotection. Firstly, cannabinoids regulate the release of retinal neurotransmitters by acting on ionic channels or enzymatic activity and are thus able to alter the retinal signal. These results were recently supported by alterations of electroretinogram recordings in mice lacking cannabinoid receptors CB1 and CB2 [51]. Unfortunately, only one study has investigated the impact of cannabis use on the human retinal function [105]. Secondly, the cannabinoid system seems to play a major regulatory role in retinal synaptic plasticity, in particular during the postnatal development. As an extension of these results, it is legitimate to question the effects of prenatal cannabis exposure on the retinal development in offspring. Thirdly, using several animal models of retinal diseases, numerous studies have shown that cannabinoids could play a neuroprotective function by preventing the retinal cell death. These results can support the development of synthetic cannabinoids as new therapeutic strategies to prevent and treat retinal diseases.

The retina constitutes a relevant and useful site to investigate neurotransmission signaling pathways as well as CNS processes. The retinal organization in mammals is well known [20] and the retina is an accessible part of the CNS that can be evaluated with noninvasive, objective, relatively rapid, and costless methods. Furthermore, its measure is quite standardized allowing good reproducibility [97, 101, 104, 106]. Functional and structural measurements allow the correlation between structural abnormalities and functional deficits. As the cannabinoid system is involved in CNS processes, the retina therefore represents a useful tool to evaluate the CNS pathophysiology and might eventually also serve to monitor curative and preventive treatment efficiency for CNS disorders.

## 7. Conclusion

This review gives an overview of the distribution of the cannabinoid system in the retina together with its involvement in the regulation of retinal neurotransmission, neuroplasticity, and neuroprotection. These suggest potential alterations of structural and functional retinal properties by exogenous cannabinoids, especially THC and cannabidiol contained in joints. As cannabis is widely spread worldwide, it is now critical to explore the effects of cannabis on the human retina. Based on experimental studies in animals, this review also aims to provide several retinal methods to correlate the cellular and molecular changes induced by cannabinoids to potential functional retinal deficits in cannabis users. However, considering the neuroprotective role of the cannabinoid system in the retina, this review also argues for therapeutic uses of synthetic cannabinoids in the treatment and the prevention of retinal diseases.
